# Comparison of EUS-guided treatment and balloon-enteroscopy-assisted endoscopic retrograde cholangiopancreatography for large bile duct stone removal in patients with surgically altered anatomy: A multicenter study

**DOI:** 10.1097/eus.0000000000000198

**Published:** 2026-07-07

**Authors:** Ryota Sagami, Kiyoyuki Kobayashi, Makoto Hinokuchi, Kosuke Takahashi, Eisuke Ozawa, Takehiko Koga, Yusuke Ishida, Shotaro Kakehashi, Nao Fujimori, Yasuhisa Hiroshima, Issei Kojima, Hideki Mori, Naosuke Kuraoka, Hidefumi Nishikiori, Kazunari Murakami, Kazuhiro Mizukami

**Affiliations:** 1 Department of Gastroenterology, Faculty of Medicine, Oita University, Oita, Japan; 2 Department of Advanced Gastrointestinal Cancer Medicine, Faculty of Medicine, Oita University, Oita, Japan; 3 Union of New Investigators for the Next Generation (U-NINE), Fukuoka, Japan; 4 Division of Innovative Medicine for Hepatobiliary and Pancreatology, Faculty of Medicine, Kagawa University, Kagawa, Japan; 5 Department of Gastroenterology, Faculty of Medicine, Kagoshima University, Kagoshima, Japan; 6 Department of Gastroenterology and Hepatology, Graduate School of Biomedical Sciences, Nagasaki University, Nagasaki, Japan; 7 Department of Gastroenterology and Medicine, Fukuoka University Faculty of Medicine, Fukuoka, Japan; 8 Department of Medicine and Bioregulatory Science, Graduate School of Medical Sciences, Kyushu University, Fukuoka, Japan; 9 Department of Gastroenterology, Nakagami Hospital, Okinawa, Japan; 10 Department of Gastroenterology, Saiseikai Kawaguchi General Hospital, Saitama, Japan; 11 Department of Gastroenterology, Oita San-ai Medical Center, Oita, Japan.

**Keywords:** double-balloon enteroscopy, assisted endoscopic retrograde cholangiopancreatography, single-balloon enteroscopy, assisted endoscopic retrograde cholangiopancreatography, EUS, guided route creation, EUS-guided hepaticogastrostomy, EUS-guided hepaticojejunostomy

## Abstract

**Background and Objectives:**

Large bile duct stone removal in patients with surgically altered anatomy (SAA) is technically challenging. Although balloon-enteroscopy-assisted endoscopic retrograde cholangiopancreatography (BE-ERCP) has been widely adopted, EUS-guided treatment (EUS-T) may achieve higher stone removal rates owing to improved device accessibility after anastomosis. In this multicenter retrospective study, we aimed to compare the outcomes of EUS-T and BE-ERCP for large bile duct stones in patients with SAA.

**Methods:**

Patients with SAA who underwent EUS-T or BE-ERCP for large bile duct stones (≥12 mm) between January 2016 and February 2025 were included. The primary outcome was the complete stone removal rate. Secondary outcomes included the number of sessions, procedure time, and adverse events. Propensity score matching was applied to adjust for baseline differences between groups.

**Results:**

In total, 22 EUS-T and 87 BE-ERCP procedures were analyzed. After matching, the complete stone removal rate was significantly higher in the EUS-T group than in the BE-ERCP group (90.5% *vs.* 61.9%, *P* = 0.030), despite a greater number of sessions (2.1 *vs.* 1.2, *P* < 0.001). Mean procedure times and adverse event rates were comparable. Cumulative success significantly improved up to the third session in EUS-T (first session, 27%; third, 86%).

**Conclusion:**

EUS-T achieved a higher complete stone removal rate than BE-ERCP without a significant increase in adverse event rates, and may be a first-line treatment option for large bile duct stones in patients with SAA.

## INTRODUCTION

Bile duct stones are a globally prevalent biliary condition.^[1]^ Endoscopic retrograde cholangiopancreatography (ERCP) is the first-line treatment for bile duct stones, including common bile duct stones and hepatolithiasis.^[2,3]^ However, ERCP using a standard duodenoscope can be challenging in patients with surgically altered anatomy (SAA) due to long reconstructed intestinal tracts, anatomical deformations, and adhesions from previous surgeries.^[4]^ Therefore, balloon-enteroscopy-assisted ERCP (BE-ERCP) has been employed in patients with SAA.^[3]^ This technique has achieved relatively high technical success and acceptable adverse event rates, offering an alternative to more invasive percutaneous or surgical treatments.^[5,6]^

Another treatment option for bile duct stones in patients with SAA is stone removal using an EUS-guided treatment (EUS-T).^[7]^ EUS-guided biliary drainage (EUS-BD) was previously associated with a high incidence of adverse events.^[8,9]^ However, both the technical success rate and the incidence of adverse events have improved over time, and the procedure has become increasingly standardized.^[10,11]^ EUS-BD was originally adopted as an alternative to ERCP for malignant cases in which ERCP was challenging.^[12]^ Subsequently, its application has expanded to benign conditions, including difficult bile duct stones in patients with SAA.^[13,14]^ The complete stone removal rates achieved with BE-ERCP and EUS-BD are comparable.^[15]^

The complete removal of large stones, also classified as difficult stones, remains technically challenging even with standard ERCP. Their removal often requires specialized devices or procedures, including endoscopic papillary large balloon dilation (EPLBD), endoscopic mechanical lithotripsy (EML), or electrohydraulic lithotripsy (EHL) with peroral cholangioscopy (POCS). These techniques generally have lower success and higher adverse event rates than procedures for the removal of small stones.^[2,3,16]^ In patients with SAA, large stone removal is considered even more technically demanding. Depending on the type of endoscope used, BE-ERCP limits the insertion of devices such as EML or EHL with POCS due to mismatches between the scope’s insertion port diameter/length and the device size.^[17–19]^ Conversely, in EUS-T, once the route is established, procedures such as EML and POCS can be performed without limitation because a standard duodenoscope is employed.

No studies have directly compared the suitability of BE-ERCP and EUS-T for large stone removal in patients with SAA.

Therefore, in this large multicenter cohort study, we aimed to compare the clinical outcomes of EUS-T and BE-ERCP for the treatment of large bile duct stones in patients with SAA. We hypothesized that large stone removal using EUS-T would achieve a higher complete stone removal rate than that of BE-ERCP.

## METHODS

### Eligibility criteria

Between January 2016 and February 2025, patients with SAA who underwent endoscopic treatment for bile duct stones using either EUS-T or BE-ERCP were identified through electronic medical records at nine participating institutions. No additional patients were included thereafter. Only procedures for large bile duct stones ≥12 mm in diameter were included in this study. Consecutive sessions of each procedure were considered a series of EUS-T or BE-ERCP procedures. Patients who were converted from failed BE-ERCP to EUS-T were included in the primary analysis if the size and number of stones remained unchanged, and were treated as independent procedural series. A sensitivity analysis excluding these participants was additionally performed to resolve the potential bias introduced by the conversion from BE-ERCP to EUS-T. Patients who received repeated alternating treatments between EUS-T and BE-ERCP (*e.g.*, first treatment with EUS-T, second with BE-ERCP, and third with EUS-T), those treated using the rendezvous technique, those undergoing ongoing treatment, and those without adequate datasets were excluded.

This study was designed and conducted in accordance with the ethical guidelines of the 1975 Declaration of Helsinki (Ninth revision, 2024). It was registered in the University Hospital Medical Network Clinical Trials Registry (UMIN000058499) as a retrospective study following approval from the Institutional Review Board (IRB protocol number, 3062-D60). The need for informed consent was waived owing to the retrospective nature of the study.

### Procedure of EUS-T and BE-ERCP

All EUS-T and BE-ERCP procedures were performed by expert pancreatobiliary endoscopists, defined as those who had performed >500 ERCP procedures and >30 EUS-guided biliary drainage procedures and were certified by the relevant professional endoscopic society.

BE-ERCP was mainly performed using EI-580BT, FUJIFILM, Tokyo, Japan, and SIF-H290S, OLYMPUS, Tokyo, Japan. EUS-HGS was mainly performed using UCT260, OLYMPUS, Tokyo, Japan, and EG-740UT, FUJIFILM, Tokyo, Japan, with the Ultrasonography devices (EU-ME2 PREMIER PLUS・ME3, OLYMPUS, Tokyo, Japan; SU-1/ARIETTA850, FUJIFILM, Tokyo, Japan). POCS + EHL was performed using SpyScopeDS Ⅱ with Autolith Touch Biliary EHL System, Boston Scientific, Tokyo, Japan.

**Figure 1.**
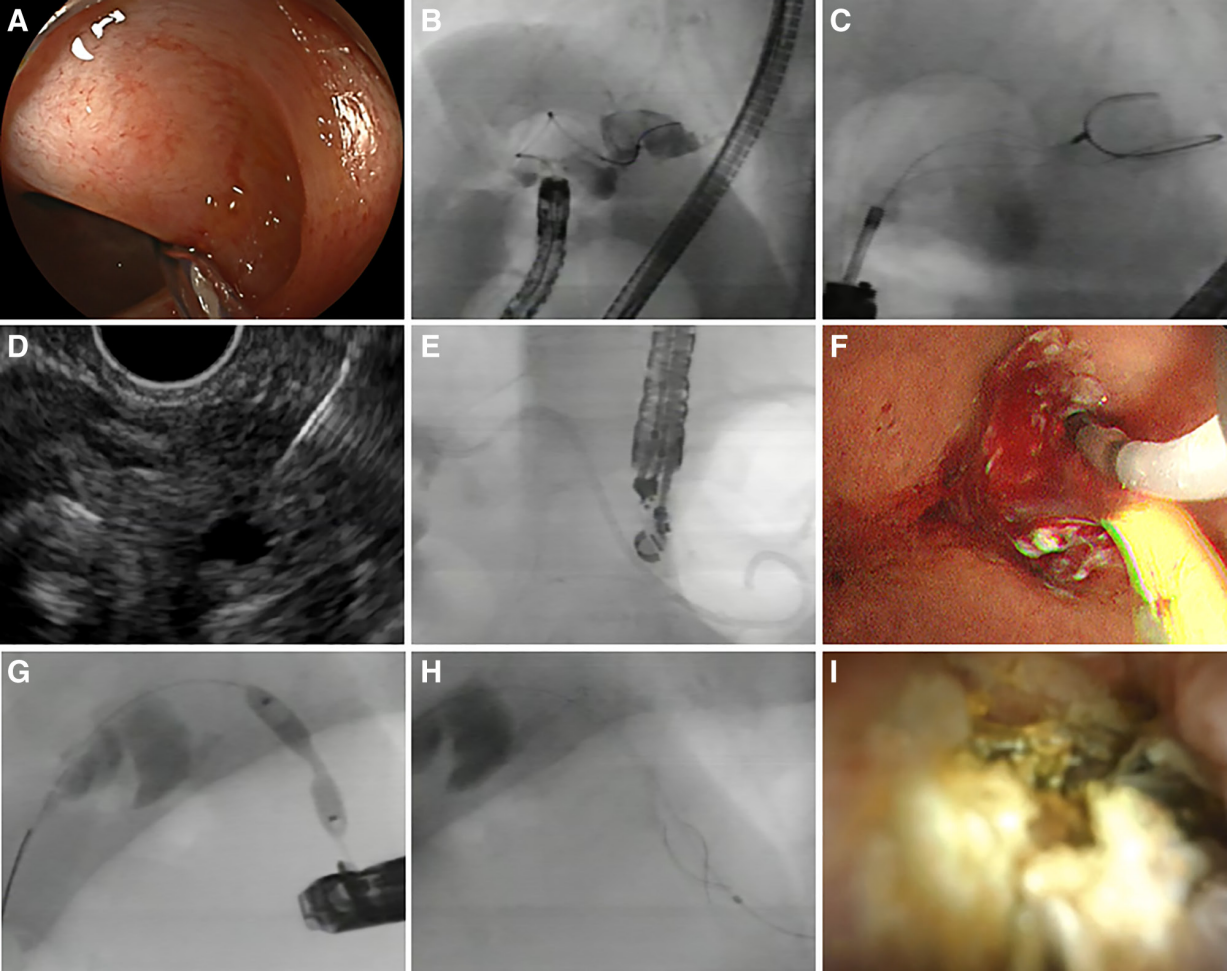
Stone removal failure with BE-ERCP and subsequent successful stone removal with EUS-T. A, Double-balloon enteroscopy was advanced to the biliary–jejunal anastomosis, and cannulation was performed. B and C, A 0.025-inch guidewire was manipulated into the peripheral bile duct across a large intrahepatic stone; however, the EML could not be inserted or used to capture the stone. D, In the subsequent EUS-T procedure, an echoendoscope was inserted, and the dilated bile duct was punctured with a 19-gauge needle. E, A dedicated plastic stent was primarily placed from the left intrahepatic bile duct to the gastric lumen. F, After several weeks, a guidewire was inserted alongside the stent through the created anastomosis. G, and H, The anastomosis was then dilated using a balloon dilation catheter; however, as it was deemed insufficiently dilated, an additional self-expandable metal stent was placed. I, Finally, stone fragmentation and removal were performed using EHL under POCS guidance. EHL, electrohydraulic lithotripsy; EML, endoscopic mechanical lithotripsy; EUS-T, EUS-guided treatment; POCS, peroral cholangioscopy.

In the BE-ERCP procedure, either single- or double-balloon enteroscopy was advanced to the papilla of Vater or the biliary–jejunal anastomosis. Cannulation was performed using a 0.025-inch guidewire. Following treatment of the papilla, such as sphincterotomy or EPLBD in selected cases, stone removal, including lithotripsy, was attempted (Figure 1A–C).

In the EUS-T procedure, an echoendoscope was inserted, and the dilated bile duct was punctured with a 19- or 22-gauge needle under Doppler ultrasound guidance to avoid vascular injury. After contrast injection, a 0.025- or 0.018-inch guidewire was advanced into the left intrahepatic bile duct using a cannulation catheter and, when possible, further advanced through the hilar bile duct into the duodenum. Antegrade stone removal was then attempted if possible; otherwise, a stent, primarily a dedicated plastic stent, was placed. After several weeks, a guidewire was inserted through the created anastomosis, which was then dilated using a balloon dilation catheter, followed by stone removal, including lithotripsy. If the anastomosis was deemed insufficiently dilated, an additional self-expandable metal stent, covered by insurance, was placed. Finally, stone fragmentation and removal were performed using lithotripsy, including EHL under POCS guidance (Figure 1D, E).

### Definitions and study endpoint

In this study, large bile duct stones were defined as those with a diameter ≥12 mm based on existing guidelines that define large stones as ≥10 or 15 mm, and this corresponds to the minimum balloon size used in EPLBD (≥12 mm) (American Gastroenterological Endoscopy Society and European Society of Gastrointestinal Endoscopy guidelines). Complete stone removal, defined as the absence of residual stones, was confirmed by cholangiography or POCS.

Procedure time was defined as the duration from scope insertion to removal. Adverse events and their severity were evaluated according to the lexicon of the American Society for Gastrointestinal Endoscopy.^[20]^ Bile peritonitis was defined as abdominal pain accompanied by elevated inflammatory markers and signs of localized inflammation, such as increased fat stranding around the procedure site on computed tomography. Procedure-related adverse events were primarily evaluated within 14 days of the endoscopic procedure.

EUS-T and BE-ERCP procedures were performed in consecutive sessions, aiming to achieve complete stone removal. Although the selection of the initial procedure and the decision to terminate the procedure were left to the operator’s discretion, treatment strategies were generally based on institutional protocols and the clinical condition of each patient. If the operator determined that stone removal was not feasible, the procedure was considered a failure. Procedure-related outcomes were assessed comprehensively across all sessions, including the final complete stone removal rate, number of procedural sessions, total procedure time, mean procedure time, devices used, adverse events, and the most severe adverse event observed.

Procedure-related factors were also compared after propensity score matching. Additionally, cumulative success rates per session, reasons for first-session failure, and the operator’s rationale for declaring procedural failure were analyzed in detail.

The primary endpoint was the comparison of complete stone removal rates between the EUS-T and BE-ERCP groups. Secondary endpoints included comparisons of procedure-related factors, particularly the number of sessions and adverse event rates. Detailed analyses of cumulative success rates by session and the specific procedural steps at which failures occurred were also conducted.

### Statistical analyses

Categorical variables were compared using the chi-square or Fisher exact tests, as appropriate. Continuous variables are expressed as mean ± standard deviation, depending on the normality of the distribution. Recurrence-free survival after complete stone removal was evaluated using the Kaplan–Meier method, and differences between groups were assessed using the log-rank test. Patients without recurrence were censored at the last follow-up. Propensity score matching was performed using a logistic regression model to generate propensity scores, followed by one-to-one matching without replacement between the EUS-T and BE-ERCP groups using a caliper width of 0.25. The matched pairs were included in the analysis. The variables included in the propensity score model were selected a priori based on their clinical relevance and potential confounding effects on treatment selection and procedural outcomes (age, sex, American Society of Anesthesiologists Physical Status [ASA-PS], presence of hepaticojejunostomy, maximum stone diameter, and number of stones). Standardized mean differences (SMD) were calculated to assess covariate balance before and after propensity score matching. For categorical variables with multiple levels, SMDs were calculated for each category. Variables with *P* < 0.10 in the univariate analysis were entered into the multivariate logistic regression model. Multicollinearity among candidate variables was assessed. We did not adjust for multiple comparisons in secondary outcomes, as these analyses were considered exploratory.

Cumulative success rates by session were analyzed using the McNemar test. All statistical analyses were conducted using SPSS, version 28.0 (IBM Corp., Armonk, NY, USA). Statistical significance was set at *P* < 0.05.

## RESULTS

### Procedure-related factors of EUS-T and BE-ERCP

**Figure 2.**
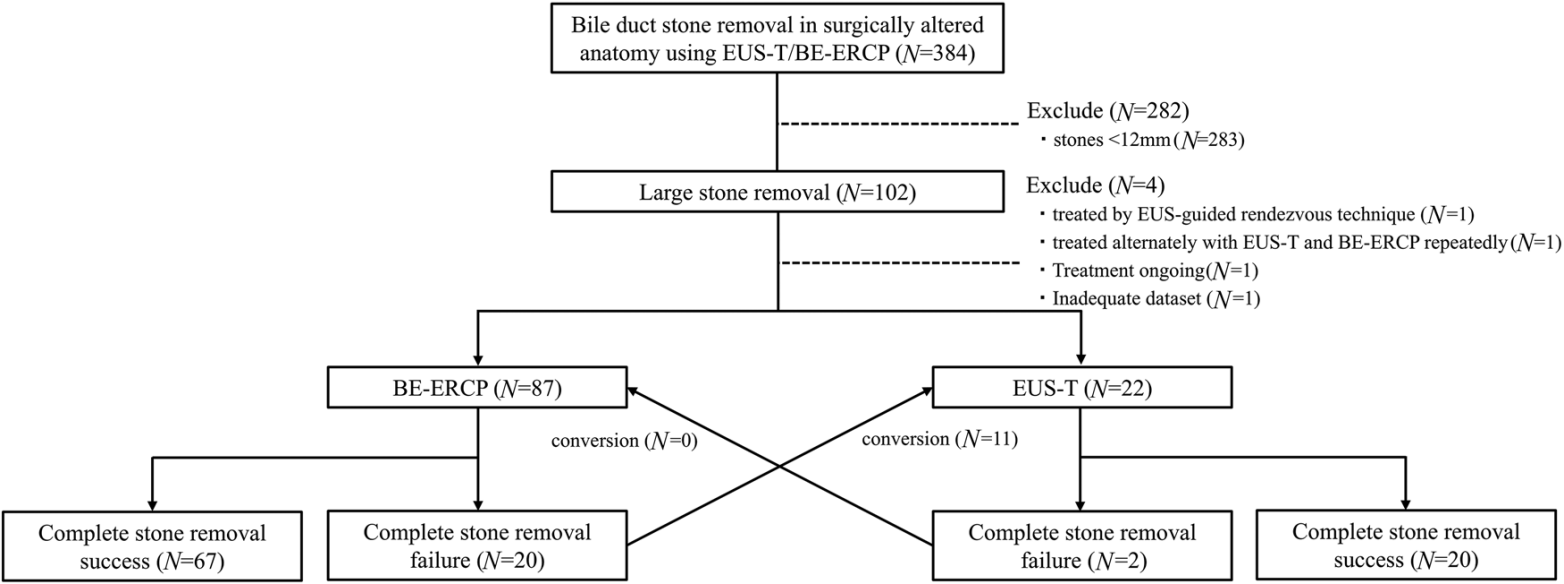
Patient flow diagram. BE-ERCP, balloon-enteroscopy-assisted endoscopic retrograde cholangiopancreatography; EUS-T, EUS-guided treatment.

Among 384 patients with SAA who underwent attempted bile duct stone removal using EUS-T or BE-ERCP, 287 were excluded due to stones <12 mm in diameter or other reasons. Eleven patients underwent conversion from BE-ERCP to EUS-T, while no patient underwent conversion from EUS-T to BE-ERCP. EUS-T and BE-ERCP procedures were evaluated as separate interventions because the size and number of target stones remained unchanged. Ultimately, 87 patients who underwent BE-ERCP and 22 who underwent EUS-T were included in the analysis (Figure 2).

Before propensity score matching, baseline characteristics between the groups were generally comparable (Table 1). The proportion of patients receiving antithrombotic therapy tended to be higher in the BE-ERCP group than in the EUS-T group. The complete stone removal rate tended to be higher in the EUS-T group than in the BE-ERCP group (90.9% *vs*. 77.0%, *P* = 0.147), although the difference was not significant (Table 2). There were no significant differences in the total number of sessions, total procedure time, or mean procedure time between the two groups. Regarding the devices used, peroral EHL with POCS and balloons were used more frequently in the EUS-T group, while baskets were used more frequently in the BE-ERCP group. There were no significant differences in Adverse events that occurred at most once during the consecutive sessions of either EUS-T or BE-ERCP.

**Table 1:** Characteristics of patients treated with EUS-T and BE-ERCP.

	EUS-T (*n* = 22)	BE-ERCP (*n* = 87)	*P* value	SMD
Age, mean (±SD), years	76.7 ± 12.7	76.0 ± 13.0	0.724	0.054
Female:male, *n* (%)	9:13	26:61	0.322	0.230
ASA-PS			0.749	
1	10 (45.5)	35 (40.2)		0.107
2	11 (50.0)	44 (50.6)		
≥3	1 (4.5)	8 (9.2)		
Charlson Comorbidity Index, mean (±SD)	3.5 ± 2.2	4.9 ± 2.5	0.758	0.594
Surgery for malignancy, *n* (%)	13 (59.1)	58 (66.7)	0.505	0.157
Primary surgery, *n* (%)			0.250	
Distal gastrectomy	8 (36.4)	36 (41.4)		0.103
Total gastrectomy	4 (18.1)	28 (32.2)		
Pancreaticoduodenectomy	2 (9.1)	7 (8.0)		
Extrahepatic bile duct resection/others	8 (36.4)	16 (18.4)		
Surgical reconstruction, *n* (%)			0.337	0.236
Roux-en-Y	17 (77.3)	58 (66.7)		
Billroth type 2/others	5 (22.7)	29 (33.3)		
Hepaticojejunostomy, *n* (%)	9 (40.9)	25 (28.7)	0.271	0.256
Time from surgery to procedures, years, mean (±SD)	19.4 ± 13.6	15.1 ± 15.1	0.810	0.299
Antithrombotic therapy, *n* (%)	1 (4.5)	20 (23.0)	0.050	0.537
Cholangitis, *n* (%)	14 (63.6)	41 (47.7)	0.181	0.320
Cholangitis, *n* (%)			0.065	
Grade 1	7 (31.8)	34 (39.1)		0.153
Grade 2	8 (36.4)	11 (12.6)		
Grade 3	0 (0)	2 (2.3)		
Stone diameter, mean (±SD)	14.6 ± 3.1	15.6 ± 5.4	0.147	0.227
The number of stone, mean (±SD)	2.3 ± 2.0	2.1 ± 1.6	0.619	0.110
Stone location, *n* (%)			0.227	
Common bile duct	15 (68.2)	73 (83.9)		0.368
Left intrahepatic duct	6 (27.3)	10 (11.5)		
Right intrahepatic duct	0 (0)	2 (2.3)		
Left and right intrahepatic duct	1 (4.5)	2 (2.3)		
Upstream bile duct diameter, mean (±SD)	13.9 ± 4.2	14.4 ± 5.1	0.657	0.107
Downstream bile duct stenosis, *n* (%)	3 (13.6)	11 (12.6)	0.901	0.030

BE-ERCP, balloon-enteroscopy-assisted endoscopic retrograde cholangiopancreatography; EUS-T, EUS-guided treatment; SD, standard deviation; SMD, standardized mean differences.

**Table 2:** Procedural factors associated with EUS-T and BE-ERCP.

	EUS-T (*n* = 22)	BE-ERCP (*n* = 87)	*P* value
Complete stone removal, *n* (%; 95% CI)	20 (90.9; 72.2–97.5)	67 (77.0; 67.1–84.6)	0.147
Complete stone removal at the first procedure time, *n* (%; 95% CI)	6 (27.3; 13.2–48.2)	40 (46.0; 35.9–56.4)	0.113
Total number of sessions, mean (±SD)	2.1 ± 1.1	1.5 ± 0.9	0.157
Total procedure time, mean (±SD) min	134.1 ± 92.6	140.1 ± 121.6	0.809
Mean procedure time, mean (±SD) min	58.8 ± 26.9	90.6 ± 36.9	0.143
Used devices, *n* (%)			
EPLBD	13 (59.1)	54 (62.1)	0.798
EML	10 (45.5)	59 (67.8)	0.052
EHL with POCS	10 (45.5)	7 (8.0)	<0.001*
Balloon	19 (86.4)	54 (62.1)	0.030*
Basket	1 (1.2)	38 (43.7)	<0.001*
Adverse events, *n* (%; 95% CI)	4 (18.2; 7.3–38.5)	12 (13.8; 8.1–22.6)	0.603
Pancreatitis	1 (1.2)	5 (5.7)	
Cholangitis	2 (9.1)	2 (2.3)	
Perforation	1 (1.2)	3 (3.4)	
Others	0 (0)	2 (2.3)	
Severity of adverse events			0.768
Slight	3 (13.6)	9 (10.3)	
Moderate	1 (1.2)	1 (1.2)	
Severe	0 (0)	1 (1.2)	
Death	0 (0)	1 (1.2)	
Recurrence of stone, *n* (%)	1 (5.0)	5 (7.4)	0.703
Follow-up time from final stone removal, mean (±SD) days	459 ± 592	852 ± 902	0.071

* indicate statistical significance.

BE-ERCP, balloon-enteroscopy-assisted endoscopic retrograde cholangiopancreatography; CI, confidence interval; EHL, electrohydraulic lithotripsy; EML, endoscopic mechanical lithotripsy; EPLBD, endoscopic papillary large balloon dilation; EUS-T, EUS-guided treatment; POCS, peroral cholangioscopy; SD, standard deviation.

Recurrence of stones occurred in 1 patient (5.0%) in the EUS-T group and 5 patients (7.4%) in the BE-ERCP group. The mean follow-up duration after complete stone removal was 459 ± 592 days in the EUS-T group and 852 ± 902 days in the BE-ERCP group (*P* = 0.071). There was no significant difference in recurrence-free survival between the groups (log-rank *P* = 0.994), and the median recurrence-free survival was not reached in either group. The mean recurrence-free survival was 2316 ± 237 days in the EUS-T group and 3064 ± 159 days in the BE-ERCP group.

In the sensitivity analysis excluding the 11 cases converted from failed BE-ERCP, the complete stone removal rate in the EUS-T group (90.9% [10/11], *P* = 0.290) remained high, and the overall trend favoring EUS-T compared with BE-ERCP was unchanged, including the adverse event rate (0% [0/11], *P* = 0.294).

Subgroup analysis according to stone size (12–15 mm *vs*. >15 mm) showed no significant differences in complete stone removal rates or adverse events between the groups in the 12–15 mm and >15 mm subgroups. Few patients underwent EUS-T in the >15 mm subgroup, so the analysis was considered exploratory.

### Analysis after propensity score matching

After propensity score matching, 21 matched pairs of EUS-T and BE-ERCP cases were compared (Table 3). Baseline characteristics between the groups were more balanced, with no significant differences. The complete stone removal rate was significantly higher in the EUS-T group than in the BE-ERCP group (90.5% *vs*. 61.9%, *P* = 0.030). However, the EUS-T group had a significantly greater total number of sessions and longer total procedure time than the BE-ERCP group (2.1 ± 1.1 *vs*. 1.2 ± 0.4 sessions and 135.1 ± 94.7 *vs*. 92.4 ± 39.9 min, respectively; both *P* < 0.001) with no significant difference in mean procedure time.

**Table 3:** Patient characteristics and procedure-related factors after propensity score matching.

	EUS-T (*n* = 21)	BE-ERCP (*n* = 21)	*P* value	SMD
Age, mean (±SD), years	78.6 ± 9.1	79.0 ± 8.1	0.369	0.046
Female:male, *n* (%)	8:13	8:13	1.000	0
ASA-PS			0.604	
1	9 (42.9)	11 (52.4)		0.190
2	11 (52.4)	8 (38.1)		
≥3	1 (4.7)	2 (9.5)		
Charlson Comorbidity Index, mean (±SD)	3.6 ± 2.1	4.1 ± 2.7	0.380	
Surgery for malignancy, *n* (%)	8 (38.1)	9 (42.9)	0.7530	.098
Primary surgery, *n* (%)			0.647	0.100
Distal gastrectomy	8 (38.1)	7 (33.3)		
Total gastrectomy	4 (19.0)	6 (28.6)		
Pancreaticoduodenectomy	2 (9.5)	3 (14.3)		
Extrahepatic bile duct resection/others	5 (23.8)	7 (33.3)		
Surgical reconstruction, *n* (%)			0.107	0.497
Roux-en-Y	16 (76.2)	11 (52.4)		
Billroth type 2/others	5 (23.8)	10 (47.6)		
Hepaticojejunostomy, *n* (%)	8 (38.1)	7 (33.3)	0.747	0.100
Time from surgery to procedures, years, mean (±SD)	18.8 ± 13.5	19.4 ± 18.2	0.232	0.037
Antithrombotic therapy, *n* (%)	1 (4.8)	4 (19.0)	0.153	0.439
Cholangitis, *n* (%)	14 (66.7)	14 (66.7)	1.000	0
Stone diameter, mean (±SD)	14.7 ± 3.2	14.4 ± 2.8	0.581	0.100
The number of stones, mean (±SD)	2.3 ± 2.1	2.0 ± 1.1	0.172	0.179
Stone location, *n* (%)			0.572	
Common bile duct	15 (71.4)	15 (71.4)		0
Left intrahepatic duct	5 (23.8)	5 (23.8)		
Right intrahepatic duct	0 (0)	1 (4.8)		
Left and right intrahepatic duct	1 (4.8)	0 (0)		
Upstream bile duct diameter, mean (±SD)	13.9 ± 4.3	12.8 ± 3.6	0.738	0.201
Downstream bile duct stenosis, *n* (%)	2 (9.5)	2 (9.5)	1.000	0
Complete stone removal, *n* (%; 95% CI)	19 (90.5; 71.1–97.3)	13 (61.9; 40.9–79.2)	0.030*	
Complete stone removal at the first procedure time, *n* (%; 95% CI)	6 (28.6; 13.8–50.0)	10 (47.6; 28.3–67.6)	0.204	
Total number of sessions, mean (±SD)	2.1 ± 1.1	1.2 ± 0.4	<0.001*	
Total procedure time, mean (±SD) min	135.1 ± 94.7	92.4 ± 39.9	<0.001*	
Mean procedure time, mean (±SD) min	59.8 ± 27.1	78.9 ± 30.2	0.593	
Adverse event, *n* (%; 95% CI)	2 (9.5; 2.7–28.9)	1 (4.8; 0.8–22.7)	0.549	
Recurrence of stone, *n* (%)	1 (4.8)	0 (0)	0.401	

* *P* < 0.005.

BE-ERCP, balloon-enteroscopy-assisted endoscopic retrograde cholangiopancreatography; CI, confidence interval; EUS-T, EUS-guided treatment; SD, standard deviation; SMD, standardized mean difference.

### Usefulness of multiple sessions of EUS-T and BE-ERCP

**Figure 3.**
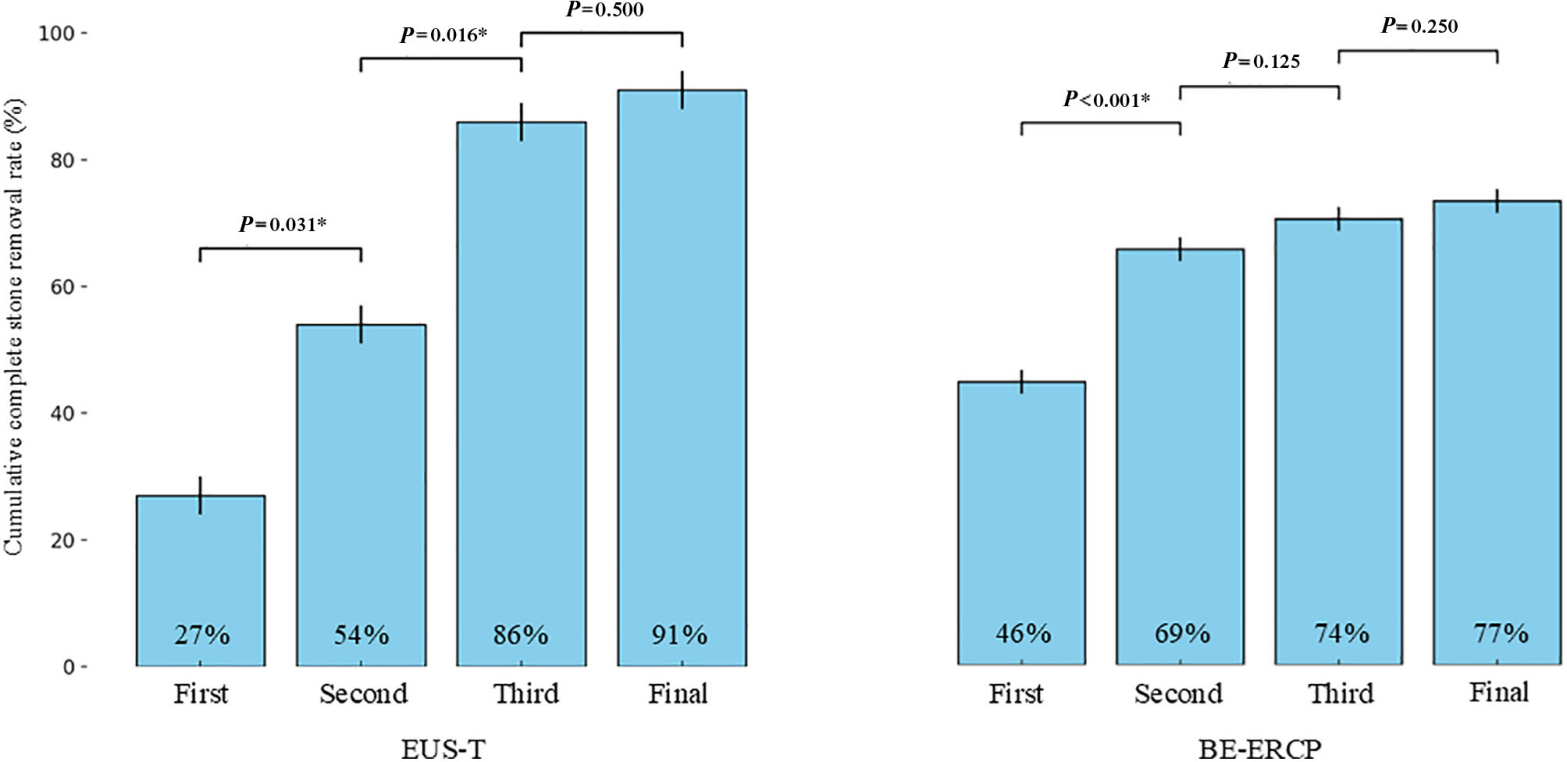
Cumulative stone removal rate with each additional session of EUS-T and BE-ERCP. BE-ERCP, balloon-enteroscopy-assisted endoscopic retrograde cholangiopancreatography; EUS-T, EUS-guided treatment. *indicate statistical significance.

Figure 3 shows the cumulative complete stone removal rate by session. In the EUS-T group, the cumulative removal rate increased significantly through the third session (first session, 27%; second, 54%; third, 86%; *P* = 0.031 and 0.016 for the second and third sessions, respectively). The cumulative rate plateaued thereafter, reaching 91% by the final session, which was not significantly different from that of the third session. Conversely, in the BE-ERCP group, the cumulative complete stone removal rate increased significantly from the first to the second session (first session, 46%; second, 69%; *P* < 0.001) but did not show further significant improvement in subsequent sessions.

In the first session of the EUS-T group, 82.4% of patients underwent only creation of the EUS-HGS route, without stone removal. In the BE-ERCP group, 53.2% of failures occurred during stone removal, 21.3% during cannulation, and 12.8% during enteroscopy insertion. Among sessions classified as procedural failure, puncture or stone removal failure was responsible in the EUS-T group (one case each), while enteroscopy insertion, cannulation, or stone removal failures accounted for most failures in the BE-ERCP group. Notably, puncture failure in the first EUS-T session and enteroscopy insertion failure in the first BE-ERCP session directly led to the decision to declare procedural failure (Table 4).

**Table 4:** Procedural steps associated with first-session failure and the session in which procedural failure was declared.

	EUS-T		BE-ERCP	n(%)
1st session	Failure cases (*n* = 17)		Failure cases (*n* = 47)	
	Puncture	1 (5.9)	Enteroscopy insertion	6 (12.8)
	Guidewire manipulation	0 (0)	Cannulation	10 (21.3)
	Only creation of EUS-HGS route	14 (82.4)	Guidewire manipulation	5 (10.6)
	Dilation	0 (0)	Dilation/sphincterotomy	1 (2.1)
	Stone removal	2 (11.8)	Stone removal	25 (53.2)
Session deciding the procedural failure	Failure cases (*n* = 2)		Failure cases (*n* = 20)	
	Puncture	1 (50.0)	Enteroscopy insertion	6 (30.0)
	Guidewire manipulation	0 (0)	Cannulation	5 (25.0)
	Only creation of EUS-BD route	0 (0)	Guidewire manipulation	3 (15.0)
	Dilation	0 (0)	Dilation/sphincterotomy	1 (5.0)
	Stone removal	1 (50.0)	Stone removal	5 (25.0)

BE-ERCP, balloon-enteroscopy-assisted endoscopic retrograde cholangiopancreatography; EUS-T, EUS-guided treatment.

### Factors affecting successful BE-ERCP

In the EUS-T procedure, the mean diameter of the punctured bile duct was 4.5 ± 1.7 mm. Hepaticojejunostomy had been performed in 18.2% of patients, and 68.2% underwent EUS-T after anastomosis maturation. As only two patients experienced incomplete stone removal, significant factors affecting procedural success could not be analyzed.

Regarding factors associated with successful BE-ERCP (Table 5), patients who achieved successful stone removal with BE-ERCP were compared with those in whom the procedure failed. Variables such as sex, ASA-PS, primary surgery, type of surgical reconstruction, presence of hepaticojejunostomy, cholangitis, stone location, type of scope used, sphincterotomy, and use of devices (EPLBD, EML, and balloon) were significant in the univariate analysis (*P* < 0.01). In the multivariate logistic regression analysis, ASA-PS and balloon use during the procedure were identified as significant factors associated with successful complete stone removal by BE-ERCP (ASA-PS: *P* = 0.044, odds ratio = 7.7, 95% confidence interval [1.053–56.333]; balloon use: *P* = 0.003, odds ratio = 21.753, 95% confidence interval [2.907–162.780]).

**Table 5:** Factors affecting successful BE-ERCP

BE-ERCP (*n* = 87)	Successful cases (*n* = 67)	Failed cases (*n* = 20)	*P* value	Multivariate logistic analysis
Age, mean (±SD), years	75.8 ± 13.4	76.7 ± 11.6	0.961	
Female:male, *n* (%)	17:50	9:11	0.092	0.890
ASA-PS			0.064	0.044*
1	23 (34.3)	12 (60.0)		
2	36 (53.7)	8 (40.0)		
≥3	8 (12.0)	0 (0)		
Charlson Comorbidity Index, mean (±SD)	5.3 ± 2.3	3.3 ± 2.4	0.300	
Etiology of surgery, *n* (%)			0.471	
Malignant	46 (68.7)	8 (40.0)		
Benign	21 (31.3)	12 (60.0)		
Primary surgery, *n* (%)			0.087	0.991
Total gastrectomy	23 (34.3)	5 (25.0)		
Distal gastrectomy	30 (44.8)	6 (30.0)		
Pancreaticoduodenectomy	6 (9.0)	1 (5.0)		
Extrahepatic bile duct resection	7 (10.4)	7 (35.0)		
Others	1 (1.5)	1 (5.0)		
Surgical reconstruction, *n* (%)			0.021*	0.776
Billroth type 2	17 (25.4)	0 (0)		
Roux-en-Y	43 (64.2)	15 (75.0)		
Others	7 (10.4)	5 (25.0)		
Hepaticojejunostomy, *n* (%)	16 (23.9)	9 (45.0)	0.067	0.783
Time from surgery to procedures, years, mean (±SD)	14.6 ± 15.6	16.8 ± 13.5	0.660	
Antithrombotic therapy, *n* (%)	1 (1.5)	4 (20.0)	0.153	
Cholangitis, *n* (%)	30 (44.8)	12 (60.0)	0.065	0.965
Stone diameter, mean (±SD)	15.5 ± 4.9	16.7 ± 6.9	0.130	
The number of stones, mean (±SD)	2.2 ± 1.5	2.1 ± 1.9	0.643	
Stone location, *n* (%)			0.076	0.748
Common bile duct	60 (90.0)	13 (65.0)		
Left intrahepatic duct	5 (7.5)	5 (25.0)		
Right intrahepatic duct	1 (1.5)	1 (5.0)		
Left and right intrahepatic duct	1 (1.5)	1 (5.0)		
Upstream bile duct diameter, mean (±SD)	14.3 ± 4.0	14.8 ± 7.9	0.223	
Downstream bile duct stenosis, *n* (%)	9 (13.4)	2 (10.0)	0.685	
Total number of sessions, mean (±SD)	1.6 ± 0.9	1.3 ± 0.9	0.260	
Total procedure time, mean (±SD) min	145.2 ± 123.2	124.9 ± 117.8	0.895	
Mean procedure time, mean (±SD) min	90.1 ± 34.5	92.2 ± 44.9	0.120	
Scope, *n* (%)			0.028*	0.906
Single balloon enteroscopy	28 (41.8)	3 (15.0)		
Double balloon enteroscopy	39 (58.2)	17 (85.0)		
Parapapillary diverticulum, *n* (%)	9 (13.4)	1 (5.0)	0.299	
Sphincterotomy, *n* (%)	16 (23.9)	1 (5.0)	0.062	0.160
Pancreatic duct guidewire insertion, *n* (%)	7 (10.4)	0 (0)	0.132	
Precut, *n* (%)	2 (3.0)	0 (0)	0.434	
Used devices use, *n* (%)				
EPLBD	48 (71.6)	6 (30.0)	<0.001*	0.182
EML	52 (77.6)	7 (35.0)	<0.001*	0.079
POCS and EHL	6 (9.0)	1 (5.0)	0.568	
Balloon	50 (74.6)	4 (20.0)	<0.001*	0.003*
Basket	33 (49.3)	5 (25.0)	0.055	0.251
AE, *n* (%)	10 (14.9)	2 (10.0)	0.837	

*indicate statistical significance. ASAP, American Society of Anesthesiologists Physical Status; BE-ERCP, balloon-enteroscopy-assisted endoscopic retrograde cholangiopancreatography; EPLBD, endoscopic papillary large balloon dilation, EML, endoscopic mechanical lithotripsy; EHL, electrohydraulic lithotripsy; POCS peroral cholangioscopy; SD, standard deviation.

## DISCUSSION

Among patients with SAA, the complete stone removal rate of large bile duct stones was higher with EUS-T than with BE-ERCP. Approximately half of the EUS-T cases were performed after failed BE-ERCP; however, sensitivity analysis excluding these cases showed similar trends. Although the difference was not significant before matching, after adjustment using propensity score matching, the complete stone removal rate was significantly higher in the EUS-T group (90.5% *vs.* 61.9%). This improvement occurred without increasing the mean procedure time or adverse event rate, despite a greater total number of sessions (2.1 ± 1.1 *vs*. 1.2 ± 0.4 sessions) and longer total procedure time (135.1 ± 94.7 *vs*. 92.4 ± 39.9 minutes).

Traditionally, percutaneous transhepatic biliary drainage or surgery has been performed for the treatment of bile duct stones in patients with SAA. However, these approaches are highly invasive and associated with a high incidence of adverse events.^[2,21,22]^ Recently, the usefulness of BE-ERCP and EUS-T has been increasingly reported, and these treatments are now being adopted as alternatives.^[6,23,24]^

Several retrospective studies have reported the clinical outcomes of EUS-T and BE-ERCP for bile duct stone removal in patients with SAA. The complete stone removal rates for EUS-T and BE-ERCP were 65.2%–87.0% and 64.5%–78.1%, respectively, with adverse event rates of 17.4%–18.6% and 7.3%–12.9%, respectively.^[22,23,25]^ A meta-analysis showed similar stone removal outcomes but highlighted the higher risk of adverse events with EUS-T.^[15]^ However, previous studies only addressed small bile duct stones measuring ≤12 mm in diameter. In this study, the severe adverse events observed in the BE-ERCP group included perforation, procedure-specific to BE-ERCP, during enteroscopy requiring emergency surgery, and death due to rupture of a hepatic artery aneurysm into the bile duct. Although the overall adverse event rates were comparable between the groups, the number of severe events was very small. Therefore, this finding may reflect the limited sample size rather than a true difference in safety profiles. Although no significant difference in recurrence-free survival was observed between the groups, the follow-up duration tended to be shorter in the EUS-T group. In addition, the mean recurrence-free survival substantially exceeded the observed follow-up duration, likely due to the high proportion of censored observations, which may have led to overestimation. Therefore, the long-term outcomes, including stone recurrence, should be interpreted with caution.

Large bile duct stones (>10–15 mm) are classified as difficult stones in clinical guidelines due to their size, which prevents removal using standard ERCP techniques employing balloons or baskets.^[2,3,16]^ Complete removal of large stones is technically difficult even in patients with normal anatomy, particularly in cases of hepatolithiasis.^[26–29]^ Procedures involving EPLBD, EML, or EHL with POCS are more technically demanding and associated with higher adverse event rates than those for smaller stones.^[2,3,16]^ SAA and large stone size are major factors that contribute to the difficulty of endoscopic treatment.^[27]^

Therefore, bile duct stones in this study may represent some of the most difficult cases, necessitating a different stone removal strategy from that used for small bile duct stones. The complete stone removal rate for EUS-T was relatively high, and that for BE-ERCP was comparable with rates reported in previous studies. Several factors may have influenced these outcomes. EUS-guided antegrade stone removal through the papilla of Vater is used for EUS-T in patients with SAA.^[22,23]^ Antegrade stone removal is performed after initial EUS-BD. However, this procedure presents technical challenges, including guidewire manipulation across the papilla, limited thrust force for antegrade extraction, difficulties using rigid EML devices, and risks of pancreatitis from papillary dilation or bile peritonitis due to an immature fistula.^[22,23,30]^ Importantly, when attempting one-step stone removal, performing the procedure before fistula maturation carries a risk of bile leakage, rendering it unsuitable for prolonged and complex interventions. However, once the EUS-guided tract has matured, transluminal stone removal becomes possible and is associated with a very low risk of pancreatitis or peritonitis.^[14,31]^ Although EUS-T via the transluminal approach has limitations, including difficulty with reintervention through the tract or stone migration into other bile ducts, a major advantage is that subsequent sessions can be performed technically more easily using a standard duodenoscope. In patients with SAA, stone removal often requires multiple sessions using either EUS-T or BE-ERCP,^[22,23,25,32]^ especially for large stones. Therefore, compared with BE-ERCP, which presents challenges in enteroscopy insertion and access, EUS-T offers a significant advantage.

EUS-T also allows full access to conventional ERCP devices, including EHL with POCS or other lithotripsy techniques. Previous case series have reported successful lithotripsy using EHL and POCS through the dilated anastomosis without adverse events.^[14]^ Additionally, fragmented stones can be removed either antegradely through the papilla into the duodenum or retrogradely through the anastomosis into the stomach. These factors likely contributed to the high complete stone removal rate observed in this study. Conversely, device availability in BE-ERCP is limited, especially with double-balloon enteroscopy.^[17–19]^ Most patients who underwent EUS-T could be treated using EML or EHL with POCS, as the use of a standard duodenoscope imposes no limitations on device selection. BE-ERCP restricted the use of these devices due to limited compatibility, particularly with double-balloon endoscopes. Although a novel slim cholangioscope that can be used during BE-ERCP has been developed, it is not yet widely available.^[33,34]^

In this study, an average of 2.1 sessions was required for EUS-T and 1.5 sessions for BE-ERCP. BE-ERCP presents procedural challenges, including scope insertion, cannulation, and stone extraction, and each session carries risks, such as perforation or pancreatitis, until complete clearance is achieved. Thus, although the mean procedure time was shorter in the EUS-T group, the difference was not significant. Conversely, EUS-T carries some risk of adverse events during the initial stent deployment, but the risks in subsequent sessions are significantly lower once the anastomosis has been created. Additionally, the overall adverse event rate between the two groups was comparable. However, severe and fatal adverse events occurred in the BE-ERCP group, including perforation. Considering complete stone removal, procedural simplicity, and safety, EUS-T may be a feasible treatment option for large bile duct stones in patients with SAA.

Moreover, the cumulative success rate for EUS-T increased up to the third session (from 27% to 86%), while that for BE-ERCP improved until the second session (from 46% to 69%). Multiple EUS-T sessions were required due to the need for anastomosis creation and additional dilation. Conversely, BE-ERCP often achieved complete stone removal within two sessions. These cumulative success rates may serve as indicators for determining procedural failure. Analysis of the procedural steps associated with failure in the first session revealed that puncture failure during EUS-T and enteroscopy insertion failure during BE-ERCP were key contributors. Accordingly, when EUS-T fails after the third session or due to puncture failure, or when BE-ERCP fails after the second session or due to insertion failure, conversion to the alternative modality should be considered.

The potential benefit of combining BE-ERCP and EUS-T in SAA has been previously discussed.^[22,24]^ In this study, 11 patients initially treated with BE-ERCP were converted to EUS-T, and the complete stone removal rate in this subgroup was 90.9%. Conversion from BE-ERCP to EUS-T based on cumulative success rates appears to be reasonable. Although no reverse conversions occurred in this cohort, considering the high complete stone removal rate for EUS-T, an alternative strategy of attempting BE-ERCP after failure of first-line EUS-T may also be considered for treating large bile duct stones in patients with SAA. Conversely, although EUS-guided biliary drainage has become more standardized and safer than in the past, it remains technically challenging, is associated with relatively high adverse event rates,^[11]^ and requires substantial experience.^[35]^ For both procedures, future efforts should focus on the standardization of strategies, broader availability of dedicated devices, and further refinement of techniques.

This is the first comparative study focusing exclusively on large bile duct stones in patients with SAA, demonstrating that EUS-T achieved a higher complete stone removal rate than BE-ERCP. However, this study has some limitations. First, the sample size was relatively small; the relatively small number of EUS-T cases may have limited the statistical power of the study, particularly for subgroup analyses. Second, the selection of the initial endoscopic procedure and the decision to terminate the procedure were left to the discretion of the operators. Additionally, approximately half of the EUS-T cases were performed after failed BE-ERCP. Although the stone size and number remained unchanged, and sensitivity analysis excluding these cases showed similar results, potential selection bias may still have influenced subsequent outcomes and generalizability. SMD after matching indicated improved covariate balance. However, some variables, including surgical reconstruction and antithrombotic therapy, remained relatively imbalanced, possibly reflecting differences in clinical decision-making during procedure selection. These residual imbalances may have introduced selection bias. Further studies stratified by surgical reconstruction and clinical background are warranted. These limitations are inherent to retrospective studies and should be addressed in future prospective or randomized controlled trials with the evaluation of long-term outcomes.

In conclusion, EUS-T may achieve a higher complete stone removal rate for large bile duct stones in patients with SAA without a significant increase in overall procedure-related adverse event rates. Therefore, it may be considered a first-line endoscopic option in experienced centers for appropriately selected patients.

## Conflicts of Interest

None.

## Source of Funding

The authors have not received any grants or funds for this research. Guarantor of the article: Ryota Sagami, the corresponding author, is accepting full responsibility for the conduct of the study.

## Ethical Statements

The study was registered in the University Hospital Medical Network Clinical Trials Registry (UMIN000058499) as a retrospective study following approval from the Institutional Review Board (IRB protocol number, 3062-D60).

## Author Contributions

R. Sagami: conceptualization, data curation, formal analysis, investigation, methodology, project administration, resources, software, supervision, validation, visualization, writing-original draft, and writing. K. Kobayashi, M. Hinokuchi, K. Takahashi, E. Ozawa, T. Koga, Y. Ishida, S. Kakehashi, N. Fujimori, Y. Hiroshima, I. Kojima, H. Mori, N. Kuraoka, and H. Nishikori: data curation, investigation, writing-original draft. K. Murakami and K. Mizukami: conceptualization, methodology, project administration, writing-review & editing. All authors reviewed and approved the article before submission.

## Data Availability Statement

The dataset used during the current study is available from the corresponding author on reasonable request.
